# Effects of In Vitro Muscle Contraction on Thermogenic Protein Levels in Co-Cultured Adipocytes

**DOI:** 10.3390/life11111227

**Published:** 2021-11-12

**Authors:** Eleni Nintou, Eleni Karligiotou, Maria Vliora, Ioannis G. Fatouros, Athanasios Z. Jamurtas, Nikos Sakellaridis, Konstantinos Dimas, Andreas D. Flouris

**Affiliations:** 1 Department of Physical Education and Sport Science, University of Thessaly, 42100 Trikala, Greece; enintou@uth.gr (E.N.); ekarligkiotou@uth.gr (E.K.); mvliora@uth.gr (M.V.); ifatouros@uth.gr (I.G.F.); ajamurt@uth.gr (A.Z.J.); 2 Department of Pharmacology, Faculty of Medicine, School of Health Sciences, University of Thessaly, 341500 Larissa, Greece; nsakella@med.uth.gr (N.S.); kdimas@med.uth.gr (K.D.)

**Keywords:** adipocytes, EPS, myotubes, thermogenesis, UCP1, muscle contraction

## Abstract

The crosstalk between the exercising muscle and the adipose tissue, mediated by myokines and metabolites, derived from both tissues during exercise has created a controversy between animal and human studies with respect to the impact of exercise on the browning process. The aim of this study was to investigate whether co-culturing of C2C12 myotubes and 3T3-L1 adipocytes under the stimuli of electrical pulse stimulation (EPS) mimicking muscle contraction can impact the expression of UCP1, PGC-1a, and IL-6 in adipocytes, therefore providing evidence on the direct crosstalk between adipocytes and stimulated muscle cells. In the co-cultured C2C12 cells, EPS increased the expression of PGC-1a (*p* = 0.129; d = 0.73) and IL-6 (*p* = 0.09; d = 1.13) protein levels. When EPS was applied, we found that co-culturing led to increases in UCP1 (*p* = 0.044; d = 1.29) and IL-6 (*p* = 0.097; d = 1.13) protein expression in the 3T3-L1 adipocytes. The expression of PGC-1a increased by EPS but was not significantly elevated after co-culturing (*p* = 0.448; d = 0.08). In vitro co-culturing of C2C12 myotubes and 3T3-L1 adipocytes under the stimuli of EPS leads to increased expression of thermogenic proteins. These findings indicate changes in the expression pattern of proteins related to browning of adipose tissue, supporting the use of this in vitro model to study the crosstalk between adipocytes and contracting muscle.

## 1. Introduction

The brite or beige adipocytes, discovered during the last decade, play a central role in the energy expenditure and the associated heat release during non-shivering thermogenesis [1,2]. Cumulating evidence is indicating the beneficial effects of beige adipocyte proliferation to the increase of insulin sensitivity as well as the reduction of circulating triglycerides and body mass index (BMI), making these cells a candidate therapeutic target in battling illnesses related to metabolism such as obesity and cardiometabolic syndrome [3,4].

White lipid cells trans-differentiate to beige adipocytes (a process known as “browning”) in response to certain types of stimuli including exposure to cold, presence of thyroid hormones and exercise, while the list of factors that can activate this mechanism is actively growing [2,5,6]. The expression of beige adipocytes specific genes and protein is commonly used to evaluate the browning capacity of adipocytes, including beige adipocytes marker protein Uncoupling Protein 1 (UCP-1), thermogenic genes Peroxisome proliferator-activated receptor γ (PPARγ), PPARγ coactivator-1 alpha (PGC-1α), PR domain containing protein 16 (PRDM16) and specific marker molecular of beige pre-adipocytes early B-cell factor 2 (EBf2) [7,8].

Recent studies showed a crosstalk between the exercising muscle and the adipose tissue, which apparently is mediated by myokines and metabolites, derived from the muscle during exercise [9,10,11]. In mice, exercise has been linked to increased mitochondrial activity, changes in gene expression, and an increase in beige adipocyte gene expression levels [12]. These studies consistently showed that exercise leads to browning of adipose tissue in mice [13,14] and that the degree of beige cell proliferation is linked with the intensity and duration of exercise [15]. However, the respective human studies show highly controversial results regarding the effectiveness of exercise/physical activity on the formation of beige adipose tissue [16,17,18]. A recent clinical trial did not detect significant browning of adipose tissue after 12 weeks of exercise [19], which is in line with several studies that have failed to identify a correlation between physical activity and browning [20,21,22]. In contrast, other studies using similar exercise protocols reported that exercise upregulated the browning process, but without any positive effects on the metabolism of the volunteers [16]. This stark controversy between animal and human studies with respect to the impact of exercise on the browning process has been demonstrated in a recent series of meta-analyses [23] and has been attributed to (i) heterogeneity of white adipose tissue depots [17], (ii) the variability in sample collection in animals and, mainly, humans [17,24], and (iii) a host of external factors (e.g., nutrition, environmental temperature, other stressors) that may interfere with the effect of exercise on the browning process [6,23]. Therefore, there is a need for in vitro experiments aiming to identify the factors and the procedures which can induce browning of white lipocytes under the effect of exercise.

Numerous studies employ the culturing of either human or mouse myotubes to identify myokines and metabolites and the use electric pulse stimulation (EPS) to mimic exercise (i.e., contraction of the myotubes) [25,26]. The electric pulse evokes contraction of the myotubes, mimicking the function of a nerve signal reaching the nerve-muscle synapse. Electrical stimulation of muscle cells in culture increases contractile properties and accelerates sarcomere assembly [27,28]. Moreover, EPS upregulates classical markers of exercise including interleukin 6 (IL-6) [29], PGC-1a [30], as well as glucose uptake [31]. While innervation and interaction with other organs is missing from in vitro exercise models [32] different protocols have managed to induce adaptations similar to resistance [32] and aerobic exercise [33]. Moreover, the in vitro contraction EPS model has proven to be a valuable tool for the identification of new myokines [25,34] as well as in the study of metabolism [35,36]. However, EPS has not been employed to date to investigate mechanisms related to tissue crosstalk in the browning process through co-culturing of different cell lines.

The aim of this study was to investigate whether co-culturing of C2C12 myotubes and 3T3-L1 adipocytes under the stimuli of EPS mimicking muscle contraction can impact the expression of UCP1, PGC-1a, and IL-6 in adipocytes, therefore providing evidence on the direct crosstalk between adipocytes and stimulated muscle cells. Based on previous findings for the use of EPS for the study of metabolic pathways [35,36,37], we hypothesized that our in vitro model of co-cultured white adipocytes and electrically stimulated myocytes to simulate exercise would increase the expression of UCP1, PGC-1a, and IL-6 in 3T3-L1 adipocytes. To our knowledge, this is the first time that the two cell types were allowed to interact in vitro under the stimuli of EPS mimicking muscle contraction, to unravel possible browning effects of contracting myotubes on adipose cells. Should our hypothesis be confirmed, this model can be used to provide precise and comprehensive mechanistic data that in vivo studies may not be able to tease apart.

## 2. Materials and Methods

### 2.1. Cell Lines and Cell Cultures

The established and well-characterized murine cell lines C2C12 and 3T3-L1 (American Type Culture Collection (ATCC, Manassas, VA, USA) were used as muscle cells and white fat lipocytes, respectively. All cells were cultured in Dulbecco’s modified Eagle’s medium (DMEM, with L-glutamine, (Gibco/BRL, Paisley, UK), 10% (*v*/*v*) fetal bovine serum (Gibco/BRL, Paisley, UK) and penicillin/streptomycin (100 IU/mL; Biosera, Ringmer, UK) at 37 °C, in an atmosphere of 5% CO_2_ in air with 100% humidity.

### 2.2. C2C12 and 3T3-L1 Differentiation Protocols

**Figure 1:**
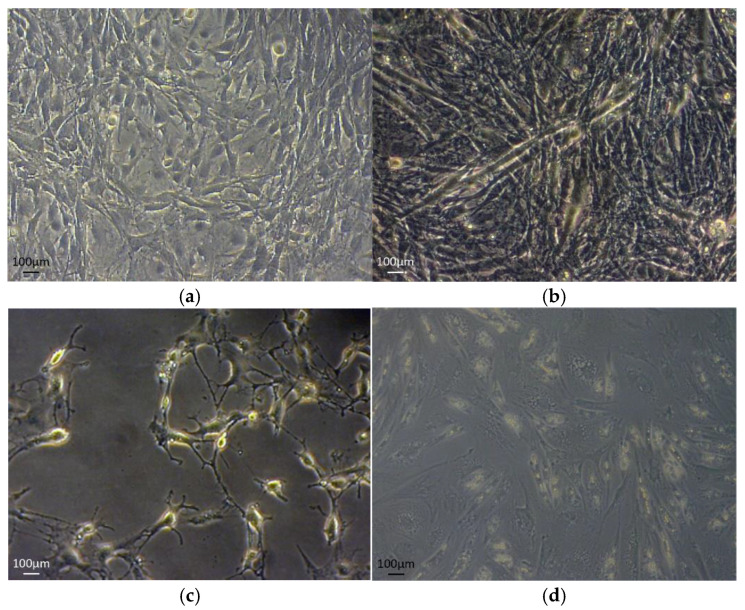
C2C12 and 3T3-L1 differentiation process. The images show undifferentiated (**a**) and differentiated (**b**) C2C12 cells, as well as undifferentiated (**c**) and differentiated (**d**) 3T3-L1 cells. All images were taken at 20× magnification.

Differentiation of C2C12 muscle cells to myotubes was achieved when at about 70–80% confluence were cultured in starvation medium of Dulbecco’s modified Eagle’s medium (DMEM, with L-glutamine) (Gibco/BRL, Paisley, UK) 2% (*v*/*v*) fetal bovine serum (Gibco/BRL, Paisley, UK) and penicillin/streptomycin (100 IU/mL; (Gibco/BRL, Paisley, UK) at 37 °C in an atmosphere of 5% CO_2_ in air with extra humidity [38]. Myotubes were formed after 5–7 days. Transformation was observed under light microscope (Figure 1a,b). All experiments were performed for passages 7–8.

Differentiation of 3T3-L1 fibroblasts was achieved by adjusting the established protocol previously described [39]. Briefly, 100% confluent cells were cultured in DMEM (Gibco/BRL, UK), 10% (*v*/*v*) fetal bovine serum (Gibco/BRL, Paisley, UK) and penicillin/streptomycin (100 IU/mL; Biosera, Ringmer, UK) supplemented with 10 μg/mL insulin (Sigma-Aldrich, Dorset, UK), 1 μM dexamethasone (Sigma, Dorset, UK) and 0.5 mM 3-isobutyl-methyl-xanthine (IBMX) (Sigma-Aldrich, Dorset, UK) medium and changed daily for 3 consecutive days, followed by a sustainability medium containing 10% FBS-DMEM containing 10 μg/mL insulin. After the course of three to five days differentiated adipocytes (accumulated lipid droplets in the cytoplasm) could be observed under the phase contrast microscope inverted Axionvert 40C) equipped with a ccd camera (Zeiss, AxionVision, software, Gottingen, Germany, accessed on 28 March 2021) until day 12 to 15 of differentiation (Figure 1c,d). All experiments were performed for passages 8–10.

### 2.3. Co–Culture Protocol

Muscle myotubes were plated in 6-well plates (SPL Life Sciences), and cells were let grow and differentiate for 6 days, while the 3T3-L1 cells were plated to grow and differentiate for 10 days, in transparent culture inserts, 0.4 μm pore size (cellQart). The inserts were hanged in the wells for the entire duration of the EPS experimental protocol [40]. 

### 2.4. EPS Protocol

**Figure 2:**
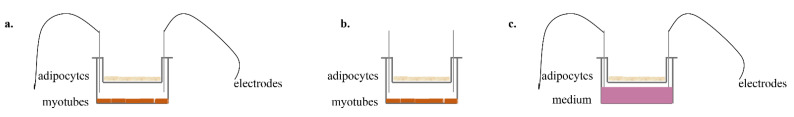
Experimental set up. Schematic representation of the three experimental conditions used: (**a**) co-culture of both cell types and EPS applied to the myotubes; (**b**) 3T3-L1 adipocytes were co-cultured with the C2C12 myotubes in absence of EPS; (**c**) EPS was applied to the well filled with medium, with only the presence of 3T3-L1 cells inserts.

The protocol was performed using a custom-made stimulator device. The EPS protocol used was an adaptation of previous established protocols [28,41,42]. Briefly, the fully differentiated C2C12 myotubes were stimulated to contract via carbon electrodes connected to the stimulator. The protocol consisted of 1 h stimulation at 50 mV/1 Hz. Three experimental conditions were established: (a) the co-culture of both cell types with EPS application to the myotubes; (b) the co-culture of both cell types without EPS application; (c) the single culture of the 3T3-L1 cells in the inserts with the EPS application on the well below, filled with medium. All cell types were harvested after 1 h rest from the EPS application (Figure 2). Independent experiments were performed at least in duplicates.

### 2.5. Lactate Dehydrogenase (LDH) Assay

For myotubes, the toxicity of EPS was determined in a colorimetric assay measuring lactate dehydrogenase (LDH) activity in the supernatant of the cell culture at the end of experiment with Cytotoxicity Detection Kit PLUS (LDH) (Roche Applied Science, Mannheim, Germany) [43] measured in a multimode plate reader (Perkin Elmer-EnSpire).

### 2.6. Western Blot Analysis

Western blot analysis was performed as described previously [44]. In short, cells were treated using lysing buffer (Biorad, Hercules, CA, USA) and the lysates were boiled in loading buffer for 10 min. Equal amounts of protein were separated by 8–12% sodium dodecyl sulphate–polyacrylamide gel and transferred onto nitrocellulose membranes ((Biorad, Hercules, CA, USA). The blots were blocked using 5% non-fat dry milk in TBS plus 0.05% Tween 20 and incubated with the primary antibody (Supplementary Table S1) overnight at 4 °C, followed by 1 h incubation at room temperature with the secondary antibody (Table S1) conjugated to horseradish peroxidase. Detection was carried out using the chemiluminescence (ECL) reaction (Biorad, Hercules, CA, USA) in Uvitec Alliance imaging system (Uvitec, Cambridge, UK). All immunoblots were performed in duplicates.

### 2.7. Statistical Analysis

Independent samples t tests and Cohen’s d effect size estimates were used to compare relative protein expression levels between co-cultured and non-co-cultured 3T3-L1 adipocytes. The same analyses were used to compare relative protein expression levels between stimulated and non-stimulated C2C12 myotubes. Given the limitations of using statistical tests based on *p* values for large and, as in this case, small sample sizes [45], the Cohen’s d effect size estimates were used to complement the *p* value comparisons, as a way to provide an additional estimate of the effect that is not based on the number of replicates. We interpreted effect sizes as small (0.2–0.5), moderate (0.5–0.8), and large (>0.80) according to Cohen’s recommendations [46]. The level of significance for the t tests was set at *p* < 0.05. All statistical analyses were performed using the statistical software package SPSS 27 for Windows (SPSS Inc., Chicago, IL, USA). The results are reported as means ± standard deviation, except otherwise indicated.

## 3. Results

### 3.1. Effects of EPS Protocol on C2C12 Myotubes

After 7 days of differentiation, the majority of the C2C12 myoblasts fused together and formed multinucleated myotubes (Figure 1). We applied EPS to differentiated C2C12 myotubes for one hour and observed by optical microscope the contraction. Differentiation and contraction of the myotubes was also confirmed by monitoring the desmin protein expression. During the treatment with EPS, no morphological changes were detected, and cell viability measured via LDH activity was not affected by the contraction protocol (Figure S1).

### 3.2. Effects of EPS Protocol on 3T3-L1/C2C12 Co-Cultured Cells

**Figure 3:**
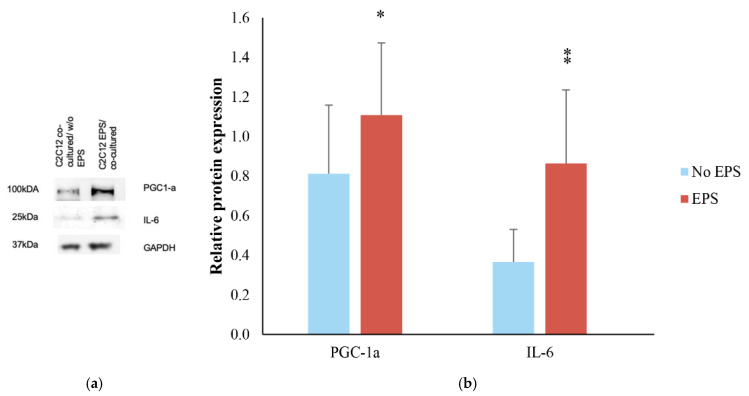
Effect of EPS and co-culture on C2C12 myotubes. (**a**) Representative blots of PGC-1a, IL-6 and GAPDH. GAPDH was used as a loading control (**b**) PGC-1a and IL-6 relative protein expression in C2C12 myotubes co-cultured with 3T3-L1 with and without EPS in relation to C2C12 differentiated and untreated cells. The band intensity was measured by densitometry and was normalized to GAPDH. All immunoblots were performed in duplicates. Graphs represent mean ± SD; asterisks indicate (*) and large (⁑) effect sizes.

Co-culturing of the 3T3-L1 cells with the C2C12 myotubes lasted for the entire contraction protocol and was followed by a resting period of one additional hour. After the resting period, PGC-1a protein levels in C2C12 myotubes showed a 1.1-fold increase and differentiated co-cultured without EPS cells had a 0.8-fold increase compared to differentiated untreated cells (Figure 3). The mean difference of PGC-1a protein levels between the two experimental conditions (with and without EPS) was 0.3 ± 0.2. The observed differences did not reach statistical significance (*p* = 0.129), yet a medium effect size was detected (d = 0.73). Similarly, IL-6 showed the same pattern of expression, presenting 0.86 times higher levels in EPS co-cultured myotubes when compared to differentiated untreated cells and 0.4-fold increase in the case of differentiated co-cultured without EPS cells. This effect of EPS did not reach statistical significance (*p* = 0.09) but showed a large effect size (d = 1.13).

**Figure 4:**
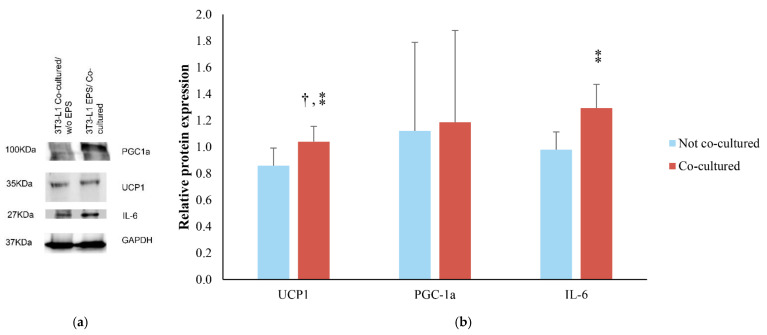
Effect of EPS and co-culture on 3T3-L1 adipocytes. (**a**) Blots of UCP1, PGC-1a, IL-6, and GAPDH. GAPDH was used as a loading control (**b**) UCP1, PGC-1a, and lL-6 relative protein expression in 3T3-L1 adipocytes with EPS co-cultured with and without C2C12 myotubes in relation to 3T3-L1 adipocytes co-cultured with C2C12 without EPS. The band intensity was measured by densitometry and was normalized to GAPDH. All immunoblots were performed in duplicates. Graphs represent mean ± SD; † indicates statistically significant difference (*p* < 0.05); ⁑ indicates large effect sizes.

The impact of the EPS contracting myotubes on the differentiated 3T3-L1 cells, when in co-culture, was examined through UCP-1, PGC-1a and IL-6 protein expression in relation to 3T3-L1 adipocytes co-cultured with C2C12 without EPS (Figure 4). UCP1 protein levels were significantly higher (*p* = 0.044) in the co-cultured adipocytes with contracted myotubes in comparison to the 3T3-L1 cells when EPS was applied without the presence of the myotubes. Moreover, this difference showed a large effect size (d = 1.29). PGC-1a was similar in EPS co-cultured cells compared to EPS treated cells (without co-culture) (*p* = 0.448; d = 0.08). Finally, IL-6 expression was higher in EPS co-cultured cells (1.3-fold increase) than EPS treated cells without co-culture (0.98-fold increase). The difference did not reach statistical significance (*p* = 0.097) but revealed a large effect size (d = 1.13).

## 4. Discussion

In this study, we let C2C12 myotubes and 3T3-L1 adipocytes interact in vitro under the stimuli of EPS, mimicking muscle contraction. We found that EPS increased the expression of PGC-1a and IL-6 protein levels in the co-cultured C2C12 cells. When EPS was applied, we found that co-culturing led to increases in UCP1 and IL-6 protein expression in the 3T3-L1 adipocytes. These findings suggest the existence of a direct crosstalk between the muscle cells, when contracted, with the adipocytes, resulting in changes in the expression pattern of proteins related to browning of adipose tissue. These findings confirm what has already been shown with in vivo rodent studies, and hence, they confirm that our model can be used in future studies to provide precise and comprehensive mechanistic data that in vivo studies may not be able to tease apart.

During and after exercise, a variety of factors are known to exert and trigger many signaling pathways, while secreted myokines have been described to have an endocrine as well as a paracrine action. Exercise-induced myokines change the profile of both muscle and adipose tissue [47]. This leads to adaptations in white adipose tissue including the reduction of the size of the adipocytes, increased mitochondrial activity, change of the adipokines profile, and changes in gene expression [12,13].

In vitro studies have investigated myokines, adipokines and metabolites that are considered modulator candidates for the formation of beige cells after exercise [9,48,49] but often without including exercise in their experimental design. In the present study, we incorporated a co-culturing of C2C12 and 3T3-L1 cells in vitro with the application of EPS on myotubes. EPS is a well-described exercise proxy that has been documented over the years as a method for inducing contraction in skeletal muscle myotubes, whereas increased levels of PGC-1a expression and other contraction related genes have been recorded [7,8]. 

The finding of elevated PGC-1a and IL-6 expression in the C2C12 myotubes after EPS application was expected, as a direct result of the contraction. The increased levels of both PGC-1a and IL-6 after electrical pulse stimulation are indicative of the activation of the metabolic adaptations in myotubes as a response to exercise, since they are known to regulate mitochondrial biogenesis [25,31,50,51]. We found that this process exerted, indirectly, an alteration in the expression of certain proteins derived by the 3T3-L1 adipocytes. Specifically, UCP1 and IL-6 production were increased after EPS induction. Moreover, the expression of PGC-1a increased by EPS but was not significantly elevated after co-culturing. This may be because PGC-1a has a relatively high turnover [52], reaching a peak expression at 15 min post stimulation, whereas our samples were assessed 60 min following EPS. When stimulated by external cues such as exercise and contraction, beige adipocytes express UCP1 protein and exhibit UCP1-dependent thermogenic capacity [24,53]. Moreover, IL-6 has a dual role: as a major myokine, it activates beige adipocyte development and is required for exercise-induced white adipose tissue browning in mice [54]; as an adipokine, IL-6 acts as a mediator in non-shivering thermogenesis and is involved in metabolic profile regulation in mice [55,56]. In line with these findings, we detected a significantly higher expression of UCP1 in 3T3-L1 adipocytes co-cultured with C2C12 myotubes under the effect of muscle contraction, in comparison to the non-co-cultured adipocytes.

A recent study investigated the effect of contracting myotubes on adipocytes where fractionated supernatant was used to culture 3T3-L1 pre-adipocytes, showing that EPS-conditioned medium promoted lipid droplet accumulation in 3T3-L1 pre-adipocytes [37]. Our experimental findings extend this work, demonstrating the existence of a direct crosstalk between the contracting muscle cells and the adipocytes, resulting in increased adipocyte expression of proteins that play key roles in the browning process (UCP-1 and IL-6).

The studied “closed”, controlled, observational in vitro system provides the advantage of having a clearer insight of the interplay between the two cell types, without the intervention of other internal or external factors. As this is the first attempt to depict this crosstalk in a closed in vitro model, through contracting myotubes co-cultured with adipocytes, these findings could ignite further research on the field. Further experimental work should also be performed with different contraction protocols as well as with the use of human myotubes and adipocytes to establish the browning effect of muscle contraction and identify the possible pathway(s) for the suggested crosstalk between cell types. There could be several hypotheses on how this communication occurs and which molecule(s) participate. This has not been elucidated by the present study and requires further investigation. This knowledge will provide a more robust theoretical framework and will, undoubtedly, increase our understanding of metabolic diseases and could be extended to understand potential connections between muscle cells and other cell types, such as cancer cells and osteocytes. Nevertheless, it is important to note that in vitro models of exercise incorporate several limitations, including lack of unanimously accepted exercise protocol characteristics and inability to study systemic effects of exercise. Consequently, results from such studies may not translate directly to whole body physiology and must be interpreted with caution and combined with in vivo studies.

The present study adopted a previously established EPS protocol [28,41,42]. Therefore, our results are limited only to this particular stimulation protocol. Future studies should test a range of muscle stimulation protocols ranging in terms of muscle stimulation characteristics (frequency, intensity, and duration). Moreover, additional browning markers, including Tbx1, Tmem26, and CD137 [57], as well as research methodologies, including gene silencing [58] and gene knock-out [59], should be investigated in future studies to confirm and expand on the present findings. Finally, it is important to note that most of our statistical comparisons based on *p* values were non-significant. Since the vast majority of our effect size comparisons were moderate or large, the lack of reaching statistical significance in most cases was not caused by increased variability, but instead, it likely reflects the small size of the data pool used in our study. As we aimed to detect biologically meaningful (instead of statistically meaningful) changes in protein expression caused by our EPS protocol, our results and conclusions are based on both *p* values and effect sizes. In recent years, many experts and scientific societies have called for complementing *p* value comparisons with other tests, such as Cohen’s effect size, to increase robustness and validity in research [45,60].

Both in vivo and in vitro models are necessary to effectively understand the mechanisms involved in the browning process. This is demonstrated by the controversy observed between in vivo human and animal studies regarding exercise-induced adipose tissue browning, which is likely caused by a variability in the exercise protocols used and different methods of browning detection [23]. Moreover, exercise exerts many whole-body adaptations, which are difficult to study separately through in vivo studies. While this can be addressed by in vitro models, previous in vitro studies did not involve exercise activity in their experimental designs [61]. This is needed to reach to a robust conclusion on the impact of exercise on the browning process. Therefore, there have been recent calls for the development of more in vitro browning models, particularly involving cell–cell signaling [62]. In the present study, the co-culturing of two different cell types under the stimulus of EPS improved previous in vitro models for studying browning because it considers, for the first time, aspects of in vivo exercise physiology. In this light, the present novel model involving contracting myocytes and adipocytes may play an important role towards the understanding of the browning process. This is particularly true since the proposed model can describe the effects of exercise on other cell types, in this case white adipocytes, which is a vital aspect of in vivo exercise physiology. However, it is important to acknowledge that in vitro models of exercise and browning, such as the present, do not account for inter-organ communication. Moreover, in vitro exercise protocols cannot be easily translated into in vivo situations [63]. Therefore, the simultaneous development of both in vivo and in vitro models that can complement each other should be employed in future studies to generate robust mechanistic data.

## 5. Conclusions

In vitro co-culturing of C2C12 myotubes and 3T3-L1 adipocytes under the stimuli of EPS leads to increased expression of thermogenic proteins. These findings support the use of the present in vitro model to study the direct crosstalk between adipocytes and contracting muscle cells which results in changes in the expression pattern of proteins related to browning of adipose tissue.

## Data Availability

The data presented in this study are available upon reasonable request from the corresponding author.

## Supplementary Materials

The following are available online at www.mdpi.com/article/10.3390/life11111227/s1, Figure S1: LDH cytotoxicity assay in C2C12 cells. Control represents medium of untreated cells. EPS treat-ment represents medium from EPS-treated cells and cytolysis describes the control for cell death, Table S1: List of antibodies used in Western blot analyses, Figure S2: Original Blots for UCP1, PGC1-a, IL-6, and GAPDH in 3T3-L1 cell line, Figure S3: Original Blots for PGC1-a, IL-6 and GAPDH in C2C12 cell line.
