# Supplementary material for: Effects of In Vitro Muscle Contraction on Thermogenic Protein Levels in Co-Cultured Adipocytes
Source: Life (Basel). 2021 Nov 12;11(11):1227. doi: 10.3390/life11111227 (PMC8625343; doi:10.3390/life11111227)
Supplement: Supplementary file 1 [file life-11-01227-s001.zip › life-1429025-supplementary.pdf]

life

MDPI

Article

Effects of In Vitro Muscle Contraction on Thermogenic Protein Levels in Co-Cultured Adipocytes

Eleni Nintou [1], Eleni Karligiotou [1], Maria Vliora [1], Ioannis G. Fatouros [1], Athanasios Z. Jamurtas [1], Nikos Sakellaridis [2], Konstantinos Dimas [2] and Andreas D. Flouris [1,*]

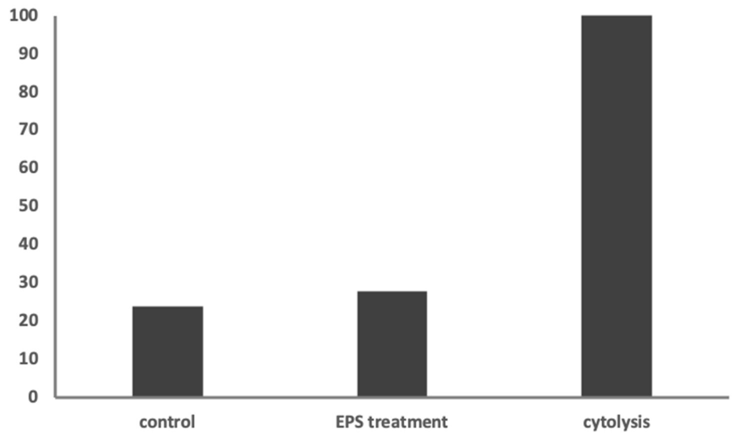

Figure S1. LDH cytotoxicity assay in C2C12 cells. Control represents medium of untreated cells. EPS treatment represents medium from EPS-treated cells and cytolysis describes the control for cell death.

Table S1. List of antibodies used in Western Blot analyses.

Antibody	Dilution	Cat.Numbers	Company
UCP1	1:1000	PA1-24894	Invitrogen
PGC-1a	1:1000	ab77210	Abcam
IL6	1:500	P620	Invitrogen
GAPDH	1:2000	AB2302	Millipore
Anti- Mouse	1:10.000	A9044	Sigma- Aldrich
Anti- Rabbit	1:10.000	7074P2	Cell Signaling

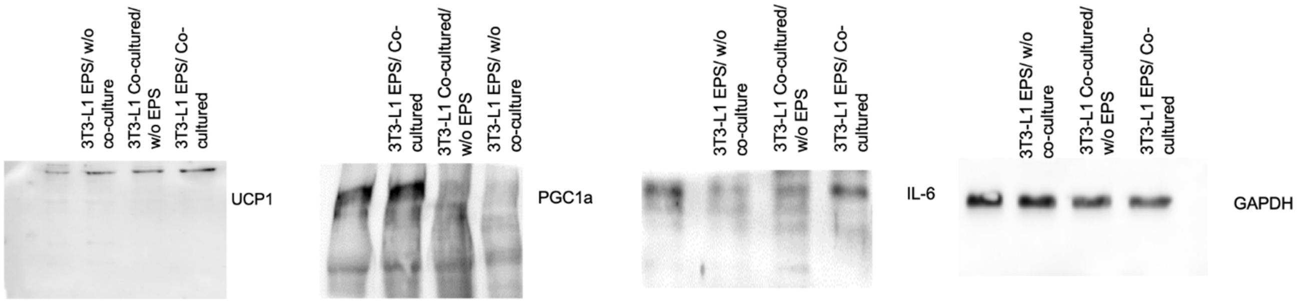

Figure S2. Original Blots for UCP1, PGC1-a, IL-6 and GAPDH in 3T3-L1 cell line.

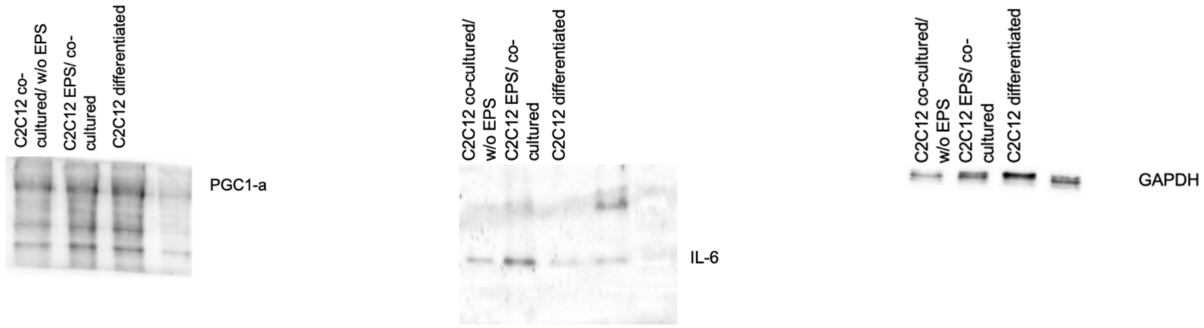

Figure S3. Original Blots for PGC1-a, IL-6 and GAPDH in C2C12 cell line.
